# Recovery of Infectious Human Norovirus GII.4 Sydney From Fomites *via* Replication in Human Intestinal Enteroids

**DOI:** 10.3389/fcimb.2021.693090

**Published:** 2021-07-07

**Authors:** Katie N. Overbey, Nicholas C. Zachos, Caroline Coulter, Joseph Jacangelo, Kellogg J. Schwab

**Affiliations:** ^1^ Department of Environmental Health and Engineering, Johns Hopkins University Bloomberg School of Public Health, Baltimore, MD, United States; ^2^ Department of Medicine, Division of Gastroenterology and Hepatology, Johns Hopkins University School of Medicine, Baltimore, MD, United States; ^3^ Stantec, Washington, DC, United States

**Keywords:** human norovirus, infectivity, human intestinal enteroids, environment, fomites, swab recovery

## Abstract

Contamination of fomites by human norovirus (HuNoV) can initiate and prolong outbreaks. Fomite swabbing is necessary to predict HuNoV exposure and target interventions. Historically, swab recovered HuNoV has been measured by molecular methods that detect viral RNA but not infectious HuNoV. The recent development of HuNoV cultivation in human intestinal enteroids (HIEs) enables detection of infectious HuNoV. It is unknown if the swabbing process and swab matrix will allow for cultivation of fomite recovered HuNoV. We used HIEs to culture swab-recovered HuNoV GII.4 Sydney from experimentally infected surfaces—a hospital bed tray (N = 32), door handle (N = 10), and sanitizer dispenser (N = 11). Each surface was swabbed with macrofoam swabs premoistened in PBS plus 0.02% Tween80. Swab eluate was tested for infectious HuNoV by cultivation in HIE monolayers. Infectious HuNoV can be recovered from surfaces inoculated with at least 10^5^ HuNoV genome equivalents/3 cm^2^. In total, 57% (N = 53) of recovered swabs contained infectious HuNoV detected by HIEs. No difference in percent positive swabs was observed between the three surfaces at p = 0.2. We demonstrate that fomite swabbing can be combined with the HIE method to cultivate high titer infectious HuNoV from the environment, filling a significant gap in HuNoV detection. Currently, high titers of HuNoV are required to measure growth in HIEs and the HIE system precludes absolute quantification of infectious viruses. However, the HIE system can provide a binary indication of infectious HuNoV which enhances existing detection methods. Identification of infectious HuNoVs from swabs can increase monitoring accuracy, enhance risk estimates, and help prevent outbreaks.

## Introduction

Human noroviruses (HuNoVs) are the leading cause of acute gastroenteritis globally and cause significant health and economic burdens (Ahmed et al., 2014). Approximately 200,000 people will die of HuNoV every year and HuNoV infections cost the global economy $64.5 billion annually (Lopman, 2015; Bartsch et al., 2016). HuNoV can be transmitted in a wide range of settings, including healthcare facilities, schools, and food service facilities (Lopman et al., 2004; Koo et al., 2009; Belliot et al., 2014; US Centers for Disease Control and Prevention, 2020).

HuNoV is spread through the fecal–oral route and virus transmission occurs from person-to-person contact, through aerosolized droplets, and from contact with contaminated fomites (Atmar and Estes, 2006). In many community settings, fomite-based transmission is of particular concern due to long environmental stability of virus particles and low viral doses required for infection (Otter et al., 2011). There is evidence that fomites can initiate HuNoV outbreaks as well as lead to longer, more severe outbreaks (Weber et al., 2010; Lopman et al., 2012; Repp and Keene, 2012; Canales et al., 2019). Swabbing of fomites is an important approach to elucidating exposure patterns (Boxman et al., 2011; Morter et al., 2011; Ronnqvist et al., 2013; Keeratipibul et al., 2017; Leone et al., 2018). Historically HuNoVs recovered from fomites have been detected by recovery of viral RNA with subsequent detection by reverse transcription-quantitative PCR (RT-qPCR) (Atmar and Estes, 2006; Knight et al., 2013). The advent of novel HuNoV culture methods offer new ways to fill important HuNoV knowledge gaps (Ettayebi et al., 2016).

In light of the important role of fomites in HuNoV transmission, numerous efforts have been undertaken to isolate and quantify HuNoV on environmental fomites. Swabbing is required to recover HuNoV from fomites and is used extensively in HuNoV outbreak investigations (Jones et al., 2007; Boxman et al., 2009). Additionally, fomite swabbing is used to identify environmental HuNoV contamination outside of outbreaks as a means to prevent transmission, monitor control efforts, and understand epidemiologic trends (Boxman et al., 2011; Morter et al., 2011; Ronnqvist et al., 2013; Keeratipibul et al., 2017; Leone et al., 2018). Swabs collected from the environment also contribute significantly to the knowledge base necessary to conduct HuNoV risk assessments (Ryan et al., 2014; Weir et al., 2016; Wilson et al., 2018). Fomite swabbing is an important laboratory technique for identifying efficacy of cleaning and disinfection protocols for HuNoV (Ciofi-Silva et al., 2019). Additionally, the International Organization for Standardization (ISO) Method 15216-1 for detection of HuNoV from foodstuffs and food surfaces specifies the need for surface swabbing (International Organization for Standardization, 2017).

Swabbing is important to HuNoV monitoring and research, but it remains imperfect, with previous reports of low and inconsistent recovery of both HuNoV and other pathogens of human health significance (Moore and Griffith, 2007; Ronnqvist et al., 2013; De Keuckelaere et al., 2014; Weir et al., 2016; Turnage and Gibson, 2017; Jones and Gibson, 2020). A number of studies have also aimed to identify the most effective methods for swab recovery of HuNoV. Though no method is 100% effective, polyurethane foam swabs pre-moistened in PBS with Tween80 appear to have relatively consistent success in recovering HuNoV and have been found to be more successful than the cotton swabs suggested by ISO methods (Moore and Griffith, 2007; Park et al., 2017; Turnage and Gibson, 2017; Jones et al., 2020). The inconsistency in swabbing literature is due to the complexity required in development of a swabbing protocol. Researchers must choose swab material, buffer composition, surface type, recovery method, and detection method to balance efficient viral recovery with logistical considerations such as short sampling time (Turnage and Gibson, 2017).

Molecular detection of HuNoV is necessitated by the historical inability to culture HuNoV in any known cell models (Duizer et al., 2004; Ettayebi et al., 2016). Molecular methods remain popular due to their high sensitivity, ease of use, and ability to provide robust quantification (Fisman et al., 2009; Knight et al., 2013). However, molecular methods are unable to distinguish infectious HuNoV particles from inactivated RNA (Knight et al., 2013). The absence of clear data on HuNoV particle infectivity hampers risk assessments, environmental monitoring, and laboratory studies of disinfection.

One method to address the lack of a readily available HuNoV cell culture model is the use of surrogate viruses. A wide range of surrogate viruses for HuNoV have been investigated, including non-human mammalian viruses and bacteriophages (Cromeans et al., 2014; Kniel, 2014). The male-specific coliphage MS2 is one of the more commonly used HuNoV surrogates due to low cost, high replication in lab settings, absence of animal pathogenicity, similarity in size and genome to HuNoV, and ease of quantification in an *E. coli* plaque assay (Bae and Schwab, 2008). MS2 has served as a valuable tool for laboratory studies of HuNoV fomite recovery and disinfection (Liu et al., 2012; Lopez et al., 2013; Tung-Thompson et al., 2015; Wengert et al., 2017). However, no surrogate is perfect, and MS2 is unlikely to accurately model HuNoV disinfection but can provide a valuable process control due to its ease of quantification in comparison to other surrogate mammalian viruses (Shirasaki et al., 2009; Solomon et al., 2009; Dunkin et al., 2017a; Dunkin et al., 2017b). Additionally, surrogates cannot fill the gap in knowledge around prevalence of infectious HuNoV that is required for robust risk assessments.

The newly developed human intestinal enteroid (HIE) model for cultivation of HuNoV offers promise in filling the gaps left by molecular detection and surrogate studies (Ettayebi et al., 2016). The HIE approach, introduced in 2016, represents the first successful attempt to culture HuNoV (Ettayebi et al., 2016). Multiple researchers have demonstrated the reproducibility of HuNoV replication in monolayers seeded from stem-cell derived HIEs (Ettayebi et al., 2016; Alvarado et al., 2018; Costantini et al., 2018; Chan et al., 2019; Koromyslova et al., 2019). The HIE method relies on measuring fold increase in viral RNA between 1 and 72 h post infection which precludes absolute quantification of viral particles (Estes et al., 2019). Due to the nature of HIE cells, no direct quantification, like that achieved with plaque assay, can occur (Ettayebi et al., 2016). Previous work has also indicated a wide variability in HuNoV replication even with consistent inputs (Costantini et al., 2018). The HIE cell model is resource and time intense, requiring multiple weeks of growth to process a single sample (Estes et al., 2019). Additionally, only some HuNoV genotypes and genogroups replicate successfully in HIEs, with reports indicating that HuNoV GII.4 shows the most successful replication (Costantini et al., 2018). Despite these challenges, HIEs remain the only way to cultivate HuNoV and offer the opportunity to address gaps in our understanding of HuNoV prevalence, risk modeling, and susceptibility to disinfectants.

Use of the HIE model to cultivate swab recovered HuNoV is necessary to measure population exposures, target areas for intervention, enhance risk assessment data, and conduct disinfection studies. However, the HIE method has not been applied to cultivation of swab recovered HuNoVs. We investigated how the complex swab matrix, which often includes salts and surfactants, impacts HuNoV GII.4 Sydney replication in HIE cells. Additionally, we determined if the process of swabbing and recovery will yield intact HuNoV GII.4 Sydney that is capable of replication in HIE cells.

## Materials and Methods

### Viral Stock Preparation

HuNoV stool suspensions were prepared from a community pediatric HuNoV case that was graciously provided by Dr. Natalie Exum. Stool was lab confirmed for HuNoV by RT-qPCR and identified as a GII.4 Sydney virus based on the capsid region (Kroneman et al., 2013). Raw stool was diluted to 10% in phosphate buffered saline (PBS) and filtered through a 0.45 µm filter. Samples were portioned and stored at −80°C from collection until time of testing. MS2 stocks were propagated and purified with an ultrafiltration membrane before portioning and storage at −80°C, as previously described (Bae and Schwab, 2008; Dunkin et al., 2017b).

### Fomite Preparation and Inoculation

**Figure 1:**
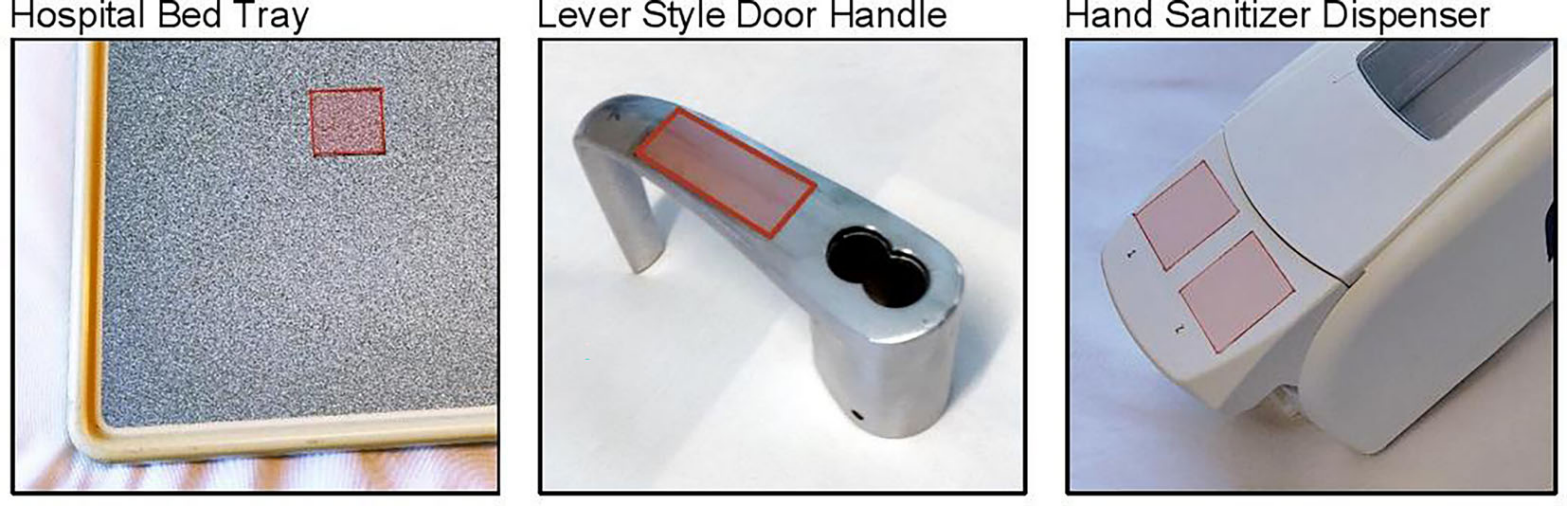
Surfaces tested for human norovirus swab recovery. Red boxes indicate 3 cm^2^ areas that were inoculated and swabbed.

Three items representing common hard, non-porous high touch fomites found in community settings were tested for HuNoV recovery in this study—a hospital bed tray (melamine-laminate), a lever-style door handle (brushed stainless steel), and a hand sanitizer dispenser [acrylonitrile butadiene styrene (ABS) plastic]. All items were kindly provided by the Johns Hopkins Hospital Facilities Management. Each item was marked with multiple 3 cm^2^ areas for sampling; swab areas were on the top surface of the bed tray, the smooth grab surface of the door handle, and the front of the push lever of the sanitizer dispenser (**Figure 1**). Prior to inoculation in all experiments, fomites were disinfected with sequential applications of 10% bleach, 70% ethanol, and distilled water. Each fomite was then exposed to a UV lamp (253.5 nm) for 30 min.

Fomite inoculum consisted of a 10% dilution of HuNoV stool suspension in PBS that ranged from 10^4^–10^6^ HuNoV genome equivalents (GE) per 3 cm^2^. When included, MS2 was added at concentrations ranging from 10^3^–10^7^ GE/3 cm^2^. First, nineteen bed tray experiments were conducted with 50µL (N = 13) or 100 µl (N = 6) of surface inoculum that included only HuNoV GII.4 Sydney. Next, ten bed tray experiments were conducted with 50 µl (N = 6) or 100 µl (N = 4) of surface inoculum with both HuNoV GII.4 Sydney and MS2 as a process control. Finally, door handle and sanitizer experiments were conducted using 50 µl of surface inoculum that contained both HuNoV and MS2.

**Figure 2:**
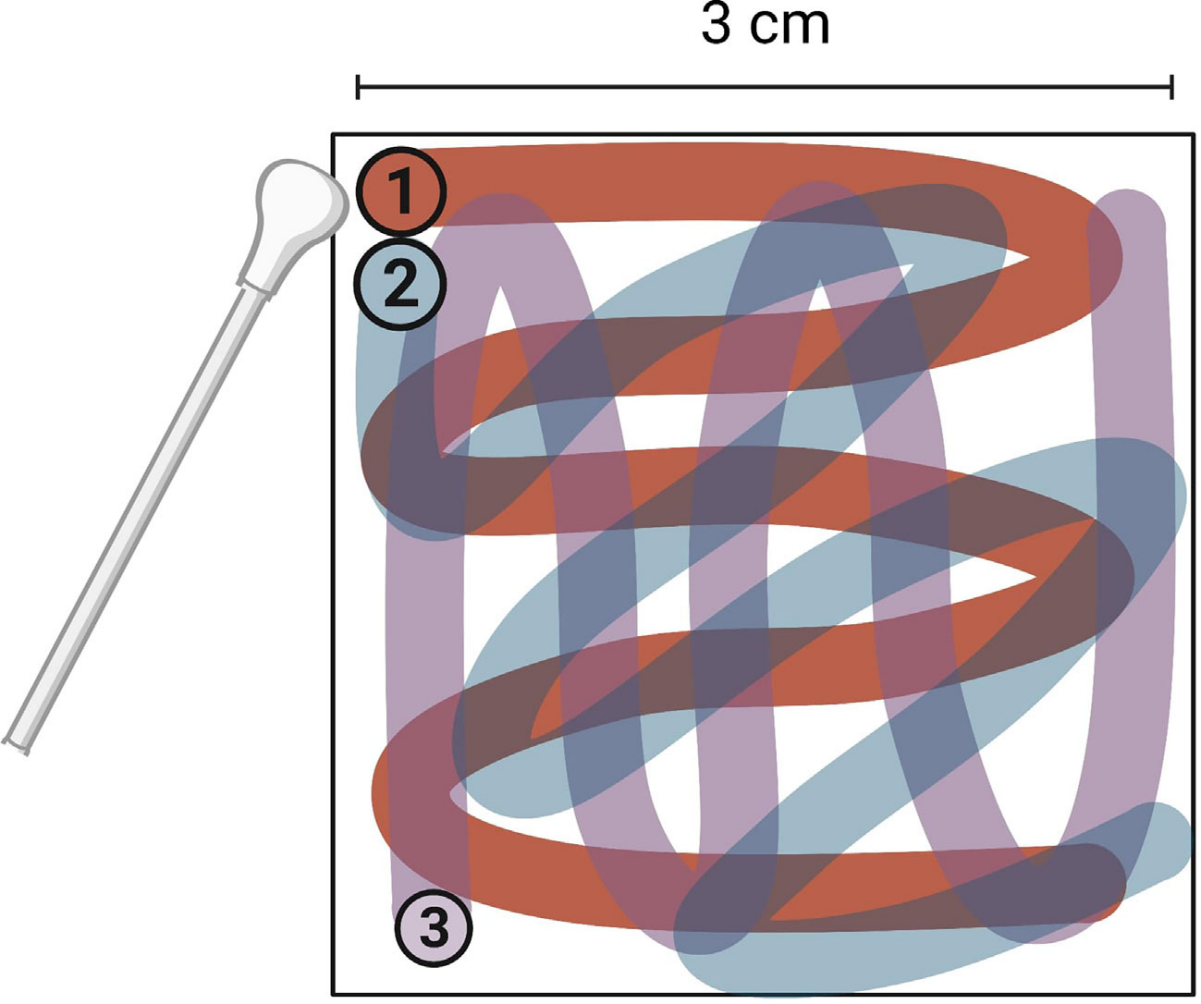
Direction and order of swabbing on surfaces tested for human norovirus swab recovery. Entire 3 cm^2^ surface was swabbed. Image created with BioRender.

Inoculation of surfaces was performed by pipetting the virus inoculum on to the surface, with the aim of covering as much of the 3 cm^2^ surface as possible and subsequently spreading the inoculum across the entire target surface. After inoculation, each fomite was immediately swabbed horizontally, vertically, and then diagonally (**Figure 2**).

### Swabbing Method

Individually wrapped, sterile 100% polyurethane foam (PUF) swabs (STX708A, Texwipe, Kernersville, NC, USA), common in industry fomite monitoring (Jones et al., 2020), were used to recover HuNoV and MS2 from inoculated fomites. Prior to swabbing, each swab was placed in a 15 ml conical tube that contained 2 ml PBS plus 0.02% Tween80 detergent for approximately 30 s to fully moisten the swab (Millipore Sigma, Burlington, MA, USA). Tween80 was added to swabbing medium as it has been shown to increase recovery of microorganisms from fomites (Moore and Griffith, 2007). Swab medium was weighed before and after swabbing to measure volume loss and calculate final eluate volume. To recover viruses, swab-containing tubes were vortexed for 30 s, centrifuged for 1 min, and then liquid was manually recovered from foam swab heads by manually pressing the swab stick along the side of the tube until no further liquid could be squeezed out. After elution, recovered swab medium was stored at −80°C until testing.

### RNA Extraction and Detection *via* RT-qPCR

Total RNA was extracted from swab eluate using Ribozol (VWR, Radnor, PA, USA) and the Direct-Zol RNA purification kit (Zymo Research, Irvine, CA, USA) as described previously (Ettayebi et al., 2016). RNA was detected and quantified using the QuantiTect Probe RT-PCR Kit (Qiagen, Hilden, Germany). Primers and probes specific to the MS2 replicase gene (Bae and Schwab, 2008) or the HuNoV ORF1-ORF2 junction (Kageyama et al., 2003) were used in the RT-qPCR assay. Final concentration for HuNoV primers was 1 and 0.2 µM for the probe. Final concentration for MS2 primers was 400 and 200nM for the probe. Thermocycler conditions for the HuNoV and MS2 RT-qPCR assays were 50°C for 30 min, 95°C for 15 min, and then 45 cycles of: 94°C for 15 s, 50°C for 15 s and 60°C for 1 min.

MS2 was quantified from molecular assays with an RNA dilution series with known amounts of coliphage. HuNoV RNA was converted from a cycle threshold value (Ct) to genome equivalents (GE) using *in vitro* RNA transcripts kindly provided by Michael Kulka (FDA, Silver Spring, MD, USA). Transcripts were derived from plasmid pNoV/MD145 which contained a full-length synthetic cDNA copy of a HuNoV GII strain (Yu et al., 2016). Molecular percent recovery was calculated by comparing HuNoV GE applied to surface to HuNoV GE in total recovered swab eluate. We confirmed the absence of RT-qPCR inhibition for HuNoV and MS2 assays with a spiked internal positive control.

### Infectivity Methods

The HIE method for culturing HuNoV has been described in detail previously (Ettayebi et al., 2016; Costantini et al., 2018). Briefly, a secretor-positive jejunal HIE cell line (J2), kindly provided by Mary Estes (Baylor College of Medicine, Houston, TX), was maintained as undifferentiated three-dimensional (3D) (i.e., spheroid) cultures embedded in Matrigel (Corning, Corning, NY, USA). HIEs were maintained at 37°C in 5% CO_2_ and Human IntestiCult media (STEMCELL Technologies, Vancouver, Canada). After 7 days of growth, 3D cultures were either passaged 1:2, archived in LiN2, or used to seed monolayers. Passaged HIE monolayers were grown for two days in IntestiCult supplemented with 10 µM Y-27632 (ROCK Inhibitor), 10 µM CHIR99021 (GSK3 inhibitor) (STEMCELL Technologies, Vancouver, Canada) and 1,000 μM/ml Primocin antimicrobial agent (InvivoGen, San Diego, CA, USA). After two days CHIR99021 was removed from the growth media.

To seed monolayers, HIEs were dissociated to a single cell suspension with Trypsin and plated 1:2 as undifferentiated monolayers in Matrigel-coated 96-well cell culture plates. Monolayers were grown for two days with IntestiCult supplemented with 10 µM Y-27632 and then differentiated for 5 days prior to infection with media lacking Wnt3a, R-spondin-1, and SB202190 (p38 MAPK inhibitor), as previously described (Saxena et al., 2016; Noel et al., 2017).

Confluent, differentiated HIE monolayers were infected apically in duplicate and all infection media was supplemented with 500 µM of glycochenodeoxycholic acid (GCDCA; Sigma-Aldrich, St. Louis, MO, USA). After 1 h of incubation at 37°C in 5% CO_2_, supernatant was removed and monolayers were washed three times with complete media without growth factors. For each set of infections, one monolayer was immediately frozen at −80°C and the second was grown at 37°C in 5% CO_2_ for 72 h post infection (hpi). Following the 72-hour incubation, the supernatant and monolayer cells were frozen at −80°C. Each monolayer experiment was conducted once per swab eluate and each set of monolayer infections included a known HuNoV GII.4 Sydney positive sample. We then extracted RNA from 1 hpi and 72 hpi monolayer cells and supernatants.

A standard 10-fold dilution, double agar plaque assay was used to enumerate infectious MS2 coliphage as plaque forming units (PFU) following the protocol described by Bae and Schwab (2008).

### Statistical Methods

Statistical analyses were performed in Stata 13 and R 3.6.1 (StataCorp, 2013; R Core Team, 2019). HuNoV replication was measured as the fold increase between HuNoV RNA copies measured at 1 and 72 hpi; samples were considered negative for replication if the fold increase was less than five. Values below the RT-qPCR LOD (44.3 RNA copies/5 µl for HuNoV and 100 RNA copies/5 µl for MS2) were replaced with the LOD value.

## Results

### Experiment Overview and Controls

Fifty individual swabbing experiments were performed: 29 on a hospital bed tray, 10 on a lever-style door handle, and 11 on a hand sanitizer dispenser. Of these 50 experiments, 30 were positive for infectious HuNoV GII.4 Sydney as measured by a 5-fold or greater increase in HuNoV RNA copies between 1 and 72 hpi in HIEs. The final HuNoV GII.4 Sydney titer at 72 hpi in HIEs after positive growth was 7.8 × 10^5^ GE/well on average and ranged from 8.7 × 10^3^ to 8.7 × 10^6^ GE/well.

Based on 14 runs of seven dilutions of RNA transcript in duplicate, the RT-qPCR limit of detection (LOD) for HuNoV was determined to be 44.3 viral RNA copies/5 µl, as calculated using the discrete threshold method (Klymus et al., 2019). The MS2 RT-qPCR limit of detection was calculated to be 100 phage RNA copies/5 µl using a dilution series of known phage stock. HuNoV infected monolayers were visually inspected under a microscope and no evidence of cytotoxic effects were observed. Known concentrations of HuNoV and MS2 RNA were spiked into negative swab and monolayer sample extracts to test for RT-qPCR inhibition. Measured RNA for spiked samples was within one CT value of known RNA concentrations (N = 5).

### Bed Tray Pilot Experiments

**Figure 3:**
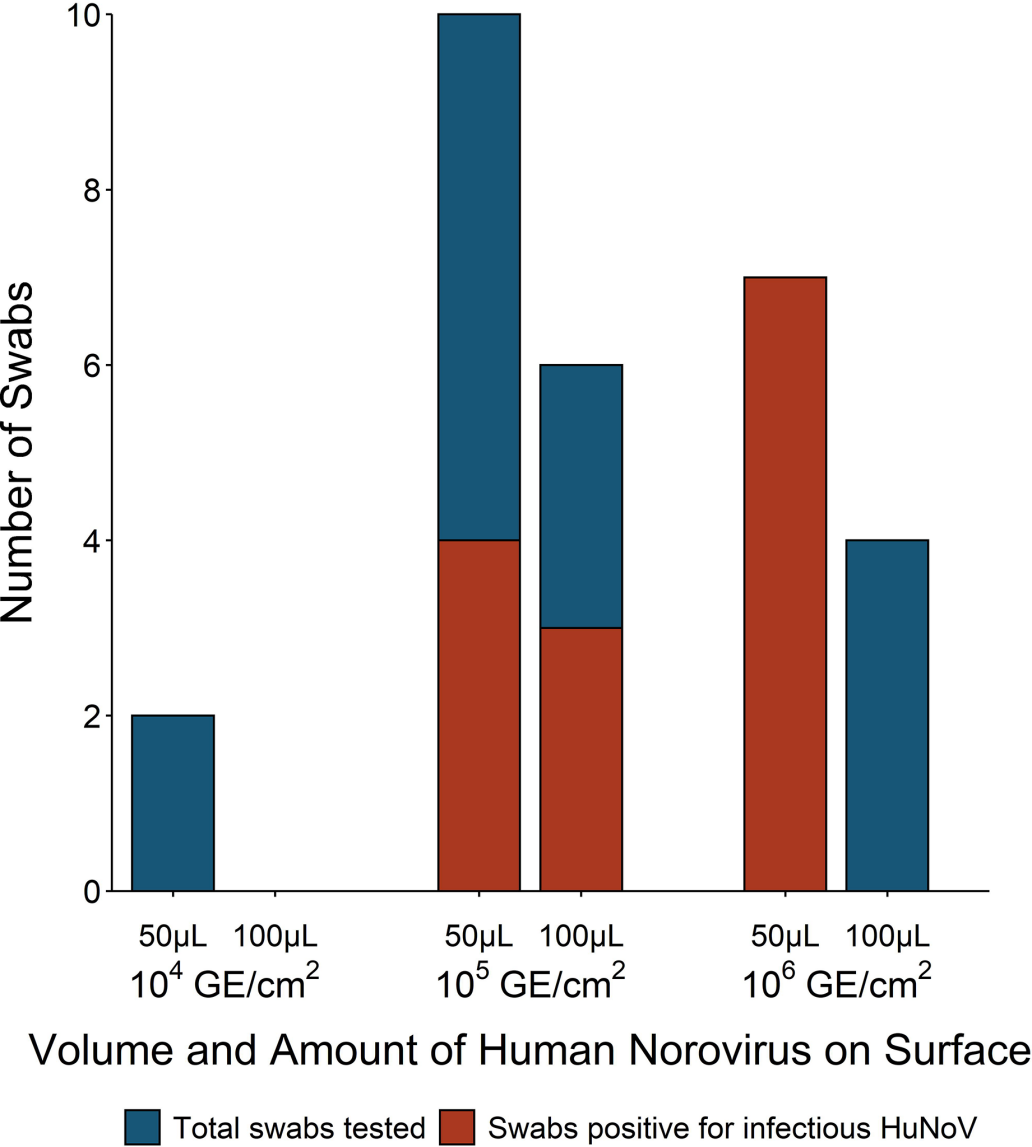
Number of fomite-recovered swabs that were tested and positive for infectious human norovirus (HuNoV) by surface inoculum volume and amount of HuNoV on fomite [genome equivalents (GE)/3 cm^2^]. Non-porous laminated fiberboard from a hospital bed tray was inoculated with either 50 or 100 ml HuNoV stool suspension containing 10^4^, 10^5^, or 10^6^ HuNoV GE/3 cm^2^. Fomites were swabbed with macrofoam swabs pre-moistened in phosphate buffered saline plus 0.02% Tween80. Human intestinal enteroid (HIE) monolayers were infected with swab eluate and were considered positive for infectious HuNoV if the fold increase in HuNoV GE between 1 and 72 h post infection (hpi) exceeded five.

Bed tray experiments were used to identify the volume and range of HuNoV GII.4 Sydney inoculum required for successful replication. Ten swabs were recovered from the bed tray after inoculation with 100 μl of HuNoV stool suspension ranging in concentration from 2.8 × 10^5^ to 2.8 × 10^6^ HuNoV GE/3 cm^2^ (**Figure 3**). Three (30%) of the 100 μl inoculum fomite-recovered swabs were positive for infectious HuNoV. Additionally, 50 μl inoculum was used in 19 bed tray swab experiments with HuNoV concentrations from 3.5 × 10^4^ to 8.7 × 10^6^ HuNoV GE/3 cm^2^ (**Figure 3**). Eleven (58%) of the 50 μl inoculum fomite-recovered swabs were positive for infectious HuNoV. The lowest fomite inoculum that resulted in recovery and detection of infectious HuNoV was 1.4 × 10^5^ GE/3 cm^2^. Less than 50% of swabs from fomites inoculated with 10^5^ HuNoV GE/3 cm^2^ contained measurable infectious HuNoV (**Figure 3**). Viral inoculum of 10^6^ HuNoV GE/3 cm^2^ or greater resulted in 0% recovery when 100 μl fomite inoculum was used and 100% recovery when 50 μl inoculum was used.

### Additional Fomite Experiments

**Figure 4:**
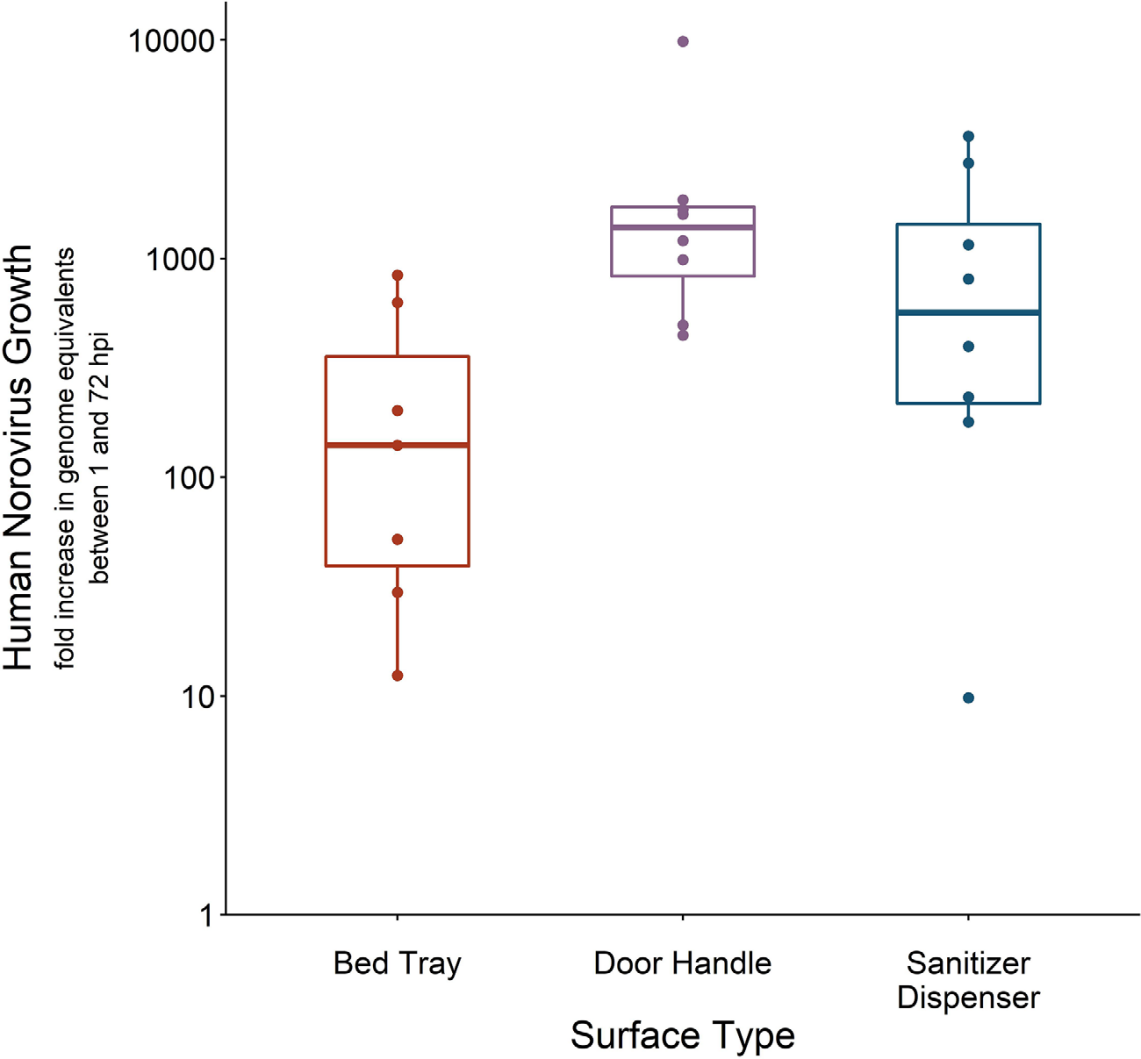
Human norovirus (HuNoV) replication from swabs recovered off lab-inoculated fomites. Three fomites—a laminated fiberboard hospital bed tray (n = 7), a brushed stainless steel door handle (n = 8), and an acrylonitrile butadiene styrene (ABS) plastic sanitizer dispenser (n = 7)—were inoculated with 50 μl HuNoV stool suspension containing 10^6^ or greater HuNoV genome equivalents (GE) per 3 cm^2^. Fomites were swabbed with macrofoam swabs pre-moistened in phosphate buffered saline plus 0.02% Tween80. Human intestinal enteroid (HIE) monolayers were infected with swab eluate and HuNoV replication is reported as the fold increase in HuNoV GE between 1 and 72 h post infection (hpi). Fold-increase of five or lower was considered negative for infectious HuNoV; only swabs positive for infectious HuNoV are shown.

Once it was established that 50 μl HuNoV stool suspension with at least 1.4 × 10^5^ HuNoV GE/3 cm^2^ led to successful recovery of infectious HuNoV from fomites, we measured recovery from metal door handles and plastic sanitizer dispensers inoculated with 10^6^ HuNoV GE/3 cm^2^. The percent of swabs that were positive for infectious HuNoV from bed tray, door handle, and sanitizer dispenser experiments were 100% (n = 7), 80% (n = 10), and 73% (n =11), respectively. Door handle-recovered swabs had the highest HuNoV replication, as measured by fold increase in HuNoV GE between 1 and 72 hpi in HIEs. The average fold increase in HuNoV GE was 2.3 × 10^3^ (SD 3.1 × 10^3^, n = 10) for door handle experiments, 1.1 × 10^3^ (SD 1.3 × 10^3^, n = 11) for sanitizer dispenser experiments, and 2.7 × 10^2^ (SD 3.3 × 10^2^, n = 7) for bed tray experiments (**Figure 4**). Recovery of infectious HuNoV was not significantly different across the three fomites when considering percent of positive swabs (ANOVA p-value = 0.5) or when considering measured fold increase (ANOVA p-value = 0.2).

### Molecular Recovery of HuNoV

**Figure 5:**
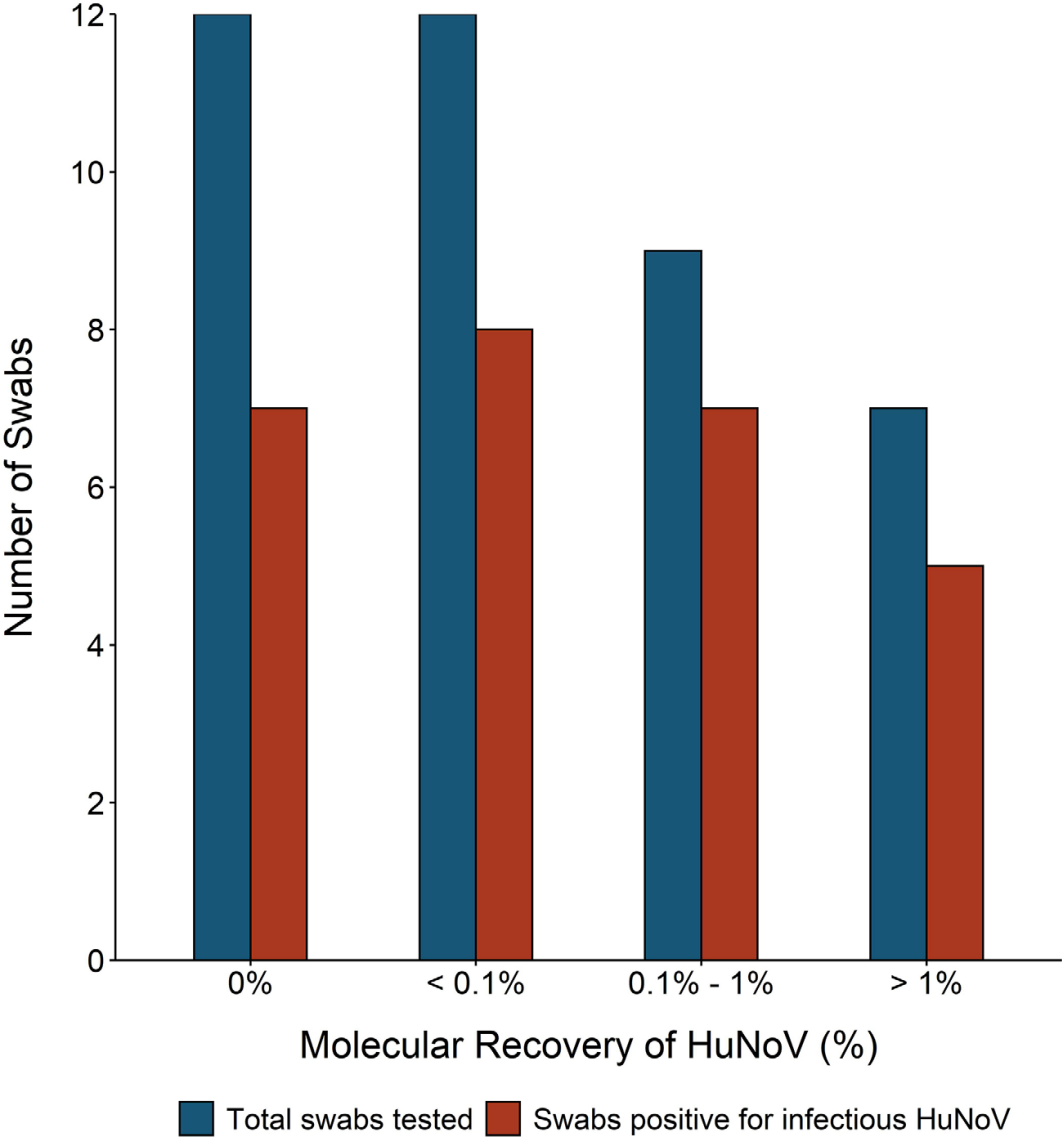
Number of swabs tested and with detectable infectious human norovirus (HuNoV) compared to molecular percent recovery of HuNoV from experimentally inoculated fomites. One of three fomites—hospital bed tray (n = 19), door handle (n = 10), or sanitizer dispenser (n = 11)—were inoculated with 50 ml of HuNoV stool suspension containing 10^4^ or greater HuNoV genome equivalents (GE) per 3 cm^2^. Fomites were swabbed with macrofoam swabs pre-moistened in phosphate buffered saline plus 0.02% Tween80. Human intestinal enteroid (HIE) monolayers were infected with swab eluate and were considered positive for infectious HuNoV if the fold increase in HuNoV GE between 1 and 72 h post infection (hpi) was greater than five. Molecular percent recovery of HuNoV was calculated by comparing HuNoV GE added to fomite to HuNoV GE in recovered eluate, using RT-qPCR.

Swabs recovered from fomites infected with 50 μl of HuNoV stool suspension were tested for molecular HuNoV recovery, in addition to HuNoV replication. Average percent recovery of HuNoV measured by molecular methods was 0.74% (range 0.03 to 4.3%) and was not significantly different across the three tested fomite types (ANOVA p-value = 0.3) (**Figure 5**). Twelve of 40 swabs had molecular HuNoV recovery below 0.1% and eight of these swabs were positive for infectious HuNoV. Percent of swabs positive for infectious HuNoV in the two higher categories of molecular recovery—0.1 to 1% and above 1%—were 77% (seven of nine) and 71% (five of seven), respectively (**Figure 5**). Twelve swabs were negative for HuNoV by molecular methods; five of these were also negative for infectious HuNoV. When controlling for HuNoV GE on fomite and fomite type, no relationship was found between detection of infectious HuNoV and molecular HuNoV percent recovery (binomial regression, all p-values >0.04).

### Recovery of MS2

**Figure 6:**
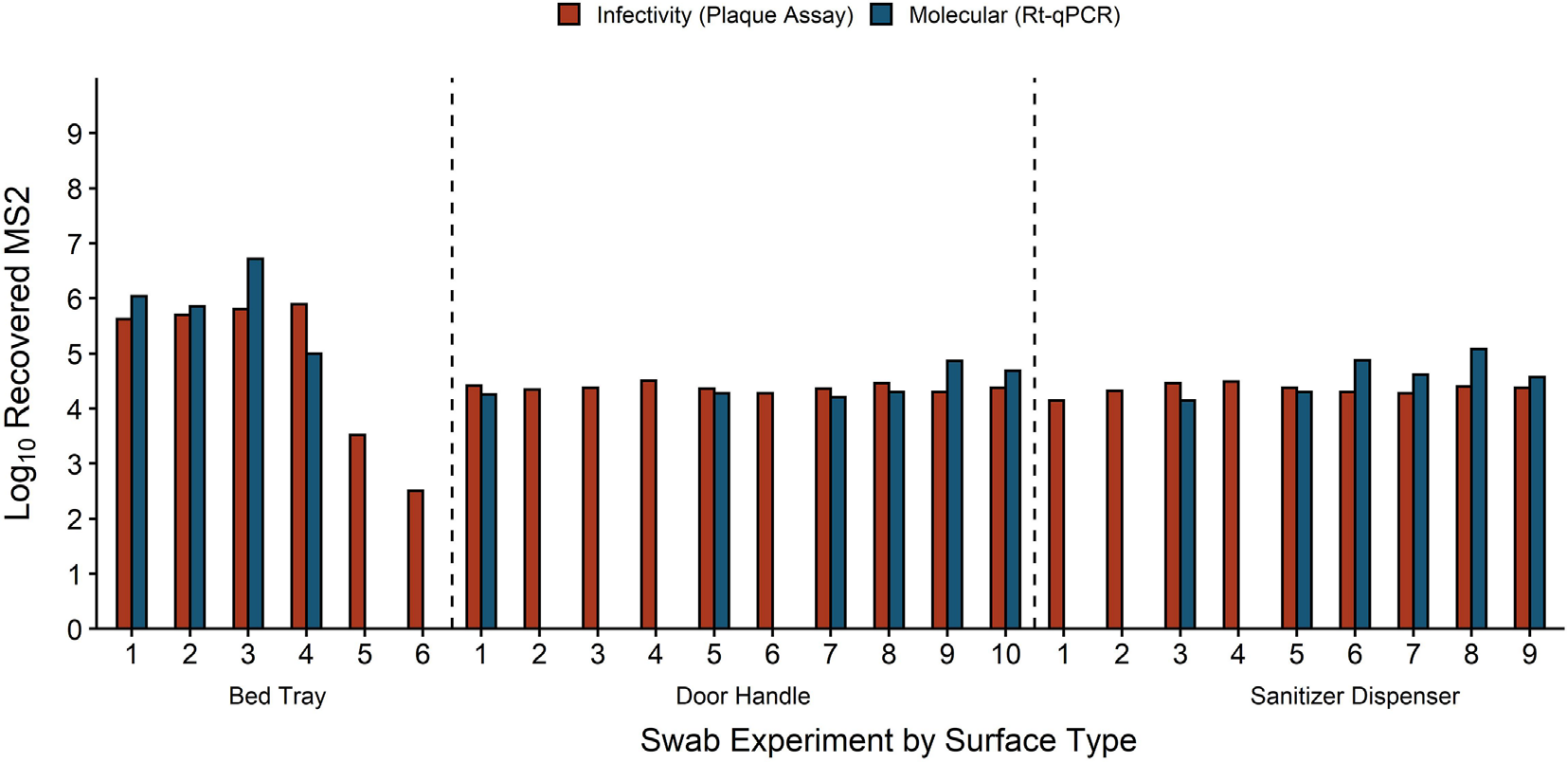
Recovery of MS2 by infectivity and molecular methods for each individual swab experiment. MS2 recovery is equivalent to plaque forming units for infectivity and genome equivalents (GE) for molecular. Fomites were inoculated with 50 μl HuNoV stool suspension mixed with 10^2^–10^7^ MS2 GE/3 cm^2^. Fomites were swabbed with macrofoam swabs pre-moistened in phosphate buffered saline plus 0.02% Tween80. Swab eluate was tested for MS2 replication with an *E. coli* plaque assay and for MS2 molecular recovery using RT-qPCR.

In addition to HuNoV stool filtrate, fomite inoculum for 25 swabbing experiments contained MS2 ranging from 1.86 × 10^2^–3 × 10^6^ GE/3 cm^2^. Infectious MS2 measured by plaque assay was found in all swab experiments run with MS2, and MS2 RNA was also detected in 18 of these swabs (**Figure 6**). Two bed tray-recovered swabs negative for MS2 RNA but not infectious MS2 were from fomites inoculated with <2 × 10^3^ MS2 GE/3 cm^2^. The other 7 swabs negative for MS2 RNA and positive for infectious MS2 were from door handle or sanitizer dispenser experiments with 3.1 × 10^4^ MS2 GE/3 cm^2^ in fomite inoculum. For samples that were positive for MS2, average percent recovery of MS2 RNA was 74% by RT-qPCR and the average percent recovery of infectious MS2 was 75% by plaque assay; no difference in either measure was found across fomite types (ANOVA p-values = 0.9 and 0.4).

## Discussion

The presence of HuNoV on fomites can inform measures of public health risk, identify targets for intervention, and indicate the efficacy of inactivation methods. The pioneering development of an HIE model for culturing infectious HuNoV promises to fill in important gaps around detection of infectious HuNoV particles after recovery from fomites (Ettayebi et al., 2016). However, the HIE model faces numerous logistical hurdles before it can be readily applied in fomite recovery research (Estes et al., 2019). This work aimed to tackle the first of these hurdles—whether swab recovered virus can successfully replicate in HIEs. We demonstrated that HuNoV GII.4 Sydney can be experimentally applied to fomites, recovered *via* swab, eluted, and subsequently replicated in HIE cell culture. Further, the data shows that the use of swab eluate comprised of PBS plus Tween80 does not lead to HIE death or prevent replication of HuNoV GII.4 Sydney in HIE culture, paving the way for use of the HIE model to cultivate swab recovered HuNoV. Our base experiments used a hospital bed tray with a smooth, laminated surface, as this type of fomite is common in health care settings where fomite swabbing is particularly necessary (Morter et al., 2011). We determined that in our experimental setup, at least 1.4 × 10^5^ HuNoV GII.4 Sydney GE/3 cm^2^ must be present on a surface to successfully recover infectious HuNoV GII.4 Sydney. Accounting for losses from swabbing, this value is consistent with previous reports of 10^3^–10^4^ GE/HIE well as a requirement for successful HuNoV replication (Ettayebi et al., 2016; Costantini et al., 2018). We also found that inoculums with equally high viral titer but applied to the surface in larger volumes (100 μl) did not result in successful recovery of infectious HuNoV GII.4 Sydney. This may be due to dilution introduced by larger volumes and the inability of the pre-moistened swab to fully recover large inoculum volumes. Importantly, HuNoVs are frequently shed at high titers and thus small droplets inoculated onto fomites can be clinically relevant (Atmar and Estes, 2006).

We first measured presence of infectious HuNoV GII.4 Sydney as binary, where a swab was considered positive if we observed a 5-fold or greater increase in GE between 1 and 72 hpi in HIEs. The number of swabs positive for infectious HuNoV was inconsistent even within equivalent surface inoculum categories, except for high viral titer (>10^6^) in 50 μl of inoculum. This indicates that high concentration viral titers provide the most successful and consistent recovery of positive virus, consistent with previous work on molecular recovery of HuNoV (Tung-Thompson et al., 2017). These values can guide future bench-scale evaluations of HuNoV fomite inactivation and having the HIE system used as a binary measure of infectious HuNoV post-fomite disinfection will be a powerful first step when developing risk models. Quantification may be possible by inoculating portions of samples in a dilution series into the HIE system, with subsequent enumeration of viral load using the most probable number (MPN) method. Another potential option for quantifying recovered infectious HuNoV is the fold increase in GE between 1 and 72 hpi in HIEs. Consistent with previous work, we found that fold increases ranged from 10–10,000 and varied from 2–3 logs within tests that used equivalent surface inoculum (Costantini et al., 2018; Chan et al., 2019; Randazzo et al., 2020). We are not the first to report high variability among measured fold increase in HIEs and this inconsistency remains a key challenge for application of the HIE system to monitoring infectious HuNoV (Costantini et al., 2018; Estes et al., 2019; Koromyslova et al., 2019).

In addition to the melamine-laminate bed tray, we tested two other fomites common in healthcare settings—a brushed stainless-steel lever-type door handle and a smooth ABS plastic sanitizer dispenser. We found no measurable differences in recovery of infectious HuNoV GII.4 Sydney off of these two fomites, compared to bed tray experiments. This is promising for future environmental monitoring work as the data suggest that multiple types of fomites can be swabbed for infectious HuNoV.

We measured molecular recovery of HuNoV GII.4 Sydney with RT-qPCR to serve as a point of comparison with infectious HuNoV data. We found that average recovery of HuNoV GII.4 Sydney from fomites as measured by molecular methods was 0.74% and ranged from 0.03 to 4.3%. These recovery values are slightly lower than most reported in the literature, which range from 4.3–100% (Scherer et al., 2009; De Keuckelaere et al., 2014; Park et al., 2015; Ibfelt et al., 2016; Tung-Thompson et al., 2017; Jones and Gibson, 2020). However, it is important to note that previously reported recovery of HuNoV from hard surfaces is highly variable both within and across studies and when reported errors are accounted for, our observations fall within previously reported ranges (Scherer et al., 2009; Ronnqvist et al., 2013; Turnage and Gibson, 2017; Jones et al., 2020). Of note, even when molecular recovery of HuNoV GII.4 Sydney was below 0.1%, approximately half of swabs were positive for infectious HuNoV. It appears that even in scenarios with low molecular recovery, infectious HuNoV particles can still be collected from fomites. We found that a few swabs were negative for HuNoV as measured by RT-qPCR, but were positive for infectious virus. This discrepancy between infectivity and molecular measures has been observed in other viruses and is likely due to methodological limitations of the RT-qPCR assay (Rose et al., 1997; Jones et al., 2009). Additionally, a smaller amount of swab eluate sample (5 µl) was tested in RT-qPCR runs, as compared to 250 µl for HIE infection, which may reduce the efficacy of RT-qPCR for low-titer samples.

As is common in HuNoV literature, we also tested the surrogate virus MS2 coliphage in a subset of swabbing experiments with both molecular and infectivity methods (Sobsey et al., 1995; Dawson et al., 2005). Recovery of MS2 *via* molecular (74%) and infectivity (75%) methods were comparable, which provides validity to our experimental set-up. However, MS2 recovery was 2 logs greater than molecular HuNoV recovery (0.74%). This recovery variation between viruses, particularly those used as surrogates, has been described in detail in the literature and in part could be due to differences in capsid structure between MS2 and HuNoV (Scherer et al., 2009; Gentry-Shields and Jaykus, 2015). Differences in virus structure can affect viral adhesion to fomites, containers, and swabs, which can then impact recovery (Langlet et al., 2007). Similar to the HIE assay, MS2 infectivity assays assess larger volumes than the volumes extracted for nucleic acid analysis which can result in positive infectivity with the absence of RT-qPCR detection. Our work adds to the extensive literature that questions the accuracy of MS2 coliphage as a HuNoV surrogate (Bae and Schwab, 2008; Richards, 2012; Knight et al., 2016; Dunkin et al., 2017b). Though MS2 retains value due to its ease of cultivation, the data from this study shows that it cannot be employed as a replacement for measuring HuNoV recovery from fomites. Thus, even when faced with multiple methodological hurdles, the HIE system has significant value as it remains the only option for specifically identifying infectious HuNoV.

This work is subject to limitations that were beyond the scope of the current study. The authors recognize the potential limitations due to evaluating three fomites, which should be considered when generalizing specific recovery measurements from this work. Additionally, we did not examine drying or viral aggregation as we were focused on confirming fomite recovery of infectious HuNoV. Though dried inoculum potentially better represents real-world fomite contamination, it was outside the scope of the reported experiments. Further, future research should consider evaluating other strains of HuNoV and additional fomite swabbing methods. Exploration of the behavior of other HuNoV genotypes and genogroups in the HIE system after fomite recovery will be necessary before this method can be used for environmental monitoring. Additional research that employs non-sterile surfaces will also be important to understand how HuNoV recovered from real-world fomites with prior surface contaminants will behave in the HIE system.

We have successfully demonstrated that the HIE culture method can be used to cultivate infectious HuNoV GII.4 Sydney recovered from fomites under prescribed conditions. This adds new utility to the HIE method and opens the door for numerous studies aimed at cultivating fomite recovered virus. Though the HIE method remains an imperfect tool, our work offers a blueprint for moving forward with fomite monitoring and disinfection studies. The most important next steps will be to address some of the hurdles that prevent wide application of the HIE system in monitoring. It will be important that future work examines factors that impact inconsistent replication of HuNoV in HIEs and aims to develop reliable methods of quantification. Additionally, reduction of the time, labor, and expense required to use HIEs for HuNoV cultivation will significantly increase the applicability of the method. The HIE method remains the only widely reproducible way to verify infectious HuNoV and our ability to recover and cultivate swab-recovered viruses moves the field one step closer to a broadly applicable system that can measure infectious HuNoV in the environment.

## Data Availability Statement

The raw data supporting the conclusions of this article will be made available by the authors upon request, without undue reservation.

## Author Contributions

All authors listed have made a substantial, direct and intellectual contribution to the work, and approved it for publication.

## Funding

KO was supported in part by a grant from the National Institutes of Health, USA (NIH grant 5T32ES007141-34). Funding was also provided by the Johns Hopkins University Education and Research Center for Occupational Safety and Health (ERC). ERC training grant funding comes from the National Institute for Occupational Safety and Health (NIOSH), under Grant No. 5 T42 OH 008428. This project was also funded through NIOSH under Grant No. R21 OH 010661. Supported in part by resources provided by NIH/NIDDK P30-DK089502 (NZ). Funding was also provided by The Osprey Foundation.

## Conflict of Interest

Author JJ was employed by company Stantec.

The remaining authors declare that the research was conducted in the absence of any commercial or financial relationships that could be construed as a potential conflict of interest.
